# Exercise induction at expression immediate early gene (c-Fos, ARC, EGR-1) in the hippocampus: a systematic review

**DOI:** 10.1590/1980-5764-DN-2023-0015

**Published:** 2024-04-15

**Authors:** Upik Rahmi, Hanna Goenawan, Nova Sylviana, Iwan Setiawan, Suci Tuty Putri, Septian Andriyani, Lisna Anisa Fitriana

**Affiliations:** 1 Universitas Pendidikan Indonesia, Department of Nursing, Bandung, West Java, Indonesia.; 2 Universitas Padjadjaran, Department of Medicine, Bandung, West Java, Indonesia.

**Keywords:** Genes, c-Fos, Hippocampus, Neurons, Exercise, Genes fos, Hipocampo, Neurônios, Exercício Físico

## Abstract

The immediate early gene exhibits activation markers in the nervous system consisting of ARC, EGR-1, and c-Fos and is related to synaptic plasticity, especially in the hippocampus. Immediate early gene expression is affected by physical exercise, which induces direct ARC, EGR-1, and c-Fos expression.

**Objective:**

To assess the impact of exercise, we conducted a literature study to determine the expression levels of immediate early genes (ARC, c-Fos, and EGR-1).

**Methods:**

The databases accessed for online literature included PubMed-Medline, Scopus, and ScienceDirect. The original English articles were selected using the following keywords in the title: (Exercise OR physical activity) AND (c-Fos) AND (Hippocampus), (Exercise OR physical activity) AND (ARC) AND (Hippocampus), (Exercise OR physical activity) AND (EGR-1 OR zif268) AND (Hippocampus).

**Results:**

Physical exercise can affect the expression of EGR-1, c-Fos, and ARC in the hippocampus, an important part of the brain involved in learning and memory. High-intensity physical exercise can increase c-Fos expression, indicating neural activation. Furthermore, the expression of the ARC gene also increases due to physical exercise. ARC is a gene that plays a role in synaptic plasticity and regulation of learning and memory, changes in synaptic structure and increased synaptic connections, while EGR-1 also plays a role in synaptic plasticity, a genetic change that affects learning and memory. Overall, exercise or regular physical exercise can increase the expression of ARC, c-Fos, and EGR-1 in the hippocampus. This reflects the changes in neuroplasticity and synaptic plasticity that occur in response to physical activity. These changes can improve cognitive function, learning, and memory.

**Conclusion:**

c-Fos, EGR-1, and ARC expression increases in hippocampal neurons after exercise, enhancing synaptic plasticity and neurogenesis associated with learning and memory.

## RESUMO.

O gene precoce imediato (GPI) exibe marcadores de ativação no sistema nervoso constituídos por ARC, EGR-1 e c-Fos e está relacionado à plasticidade sináptica, especialmente no hipocampo. A expressão do GPI é afetada pelo exercício físico, que induz a expressão direta de ARC, EGR-1 e c-Fos.

**Objetivo:**

Para avaliar o impacto do exercício físico, realizamos um estudo de literatura para determinar os níveis de expressão dos GPIs (ARC, c-Fos e EGR-1).

**Métodos:**

A base de dados utiliza literatura *on-line*, PubMed-Medline, Scopus e ScienceDirect. O artigo original em inglês usa as seguintes palavras-chave em seu título: (Exercise) AND (c-Fos) AND (Hippocampus), (Exercise) AND (ARC) AND (Hippocampus), (Exercise) AND (EGR-1) AND (Hippocampus).

**Resultados:**

O exercício físico pode afetar a expressão de EGR-1, c-fos e ARC no hipocampo, uma parte importante do cérebro envolvida na aprendizagem e na memória. O exercício físico aumenta a expressão do gene c-Fos; sua alta intensidade pode aumentar a expressão de c-Fos, indicando ativação neural. Além disso, a expressão do gene ARC aumentou devido ao exercício físico, onde ARC é um gene que desempenha um papel na plasticidade sináptica e na regulação da aprendizagem e da memória, nas mudanças na estrutura sináptica e no aumento das conexões sinápticas, enquanto o EGR-1 também desempenha um papel na plasticidade sináptica, uma mudança genética que afeta o aprendizado e a memória. De maneira geral, o exercício físico regular pode aumentar a expressão de ARC, c-fos e EGR-1 no hipocampo. Isso reflete as mudanças na neuroplasticidade e na plasticidade sináptica que ocorrem em resposta à atividade física. Essas mudanças podem melhorar a função cognitiva, o aprendizado e a memória.

**Conclusão:**

A expressão de c-Fos, EGR-1 e ARC aumenta após o exercício físico nos neurônios do hipocampo, para aumentar a plasticidade sináptica, a neurogênese associada ao aprendizado e à memória.

## INTRODUCTION

The group of genes that are activated by neurons is known as immediate early genes (IEG)^1^. IEG is essential in brain function, particularly in the synaptic process. IEG neurons are regulated by cellular and synaptic developmental responses^2^. The expression of c-Fos, ARC, and early growth response 1 (EGR-1)/zif268 represents subsets of IEG genes. These genes are rapidly and selectively controlled in hippocampal learning and memory^3^.

Numerous studies explored neural IEG^4^. One extensively studied gene is the cAMP-responsive element-binding protein (CREB), which plays a crucial role in regulating synaptic development and plasticity^5^. IEG expression is influenced by nerve stimulation; for instance, EGR-1 messenger RiboNucleic Acid (mRNA) depends on N-methyl-D-aspartate (NMDA) glutamate receptors, while c-Fos is independent of nerve stimulation^6,7^. As part of the IEG family, ARC is an effector involved in neural signaling pathways, not a transcription factor. However, the ARC gene is transcribed in response to neuronal activity and synaptic activation of neuron dendrites^8,9^.

Several IEGs play a role in encoding transcription factors and transiently enhancing transcription in the rat dentate gyrus following afferent stimulation induced by long-term potentiation (LTP) synaptic plasticity or persistent and long-term potential dependent on the activity of associative memory mechanisms. Among these genes, EGR-1/zif268 is most explicitly linked to LTP because it is induced in all LTP-triggering situations and shows a very high correlation with LTP duration^8,10^.

This review aims to elucidate the role of IEG in synaptic plasticity in exercise-induced learning and memory. While many other review articles have discussed IEG in synaptic plasticity, none have specifically delved into the role of exercise-induced IEG genes in synaptic plasticity.

## METHODS

**Figure 1:**
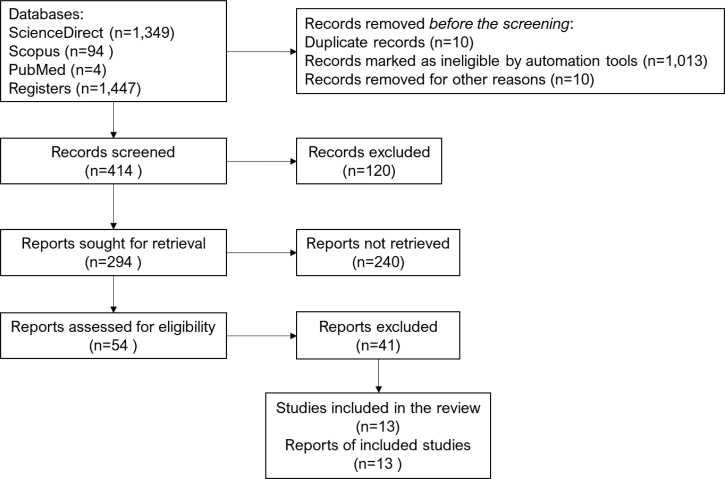
Flowchart of the process of selecting research studies that have been identified and elected based on criteria, research processes, and databases.

We conducted a comprehensive search of online literature databases, including Scopus, PubMed-Medline, and ScienceDirect. The original articles in English were selected using the following keywords in the title: (Exercise OR physical activity) AND (c-Fos) AND (Hippocampus), (Exercise OR physical activity) AND (ARC) AND (Hippocampus), (Exercise OR physical activity) AND (EGR-1 OR zif268) AND (Hippocampus) between 2013 and 2023. Our analysis was limited to experimental studies with exercise interventions, and we included articles in English published between 1987 and 2022 (Figure 1).

Studies in animals that involved exercise interventions and included neurocognitive impairment were considered. However, we excluded systematic reviews, literature articles, and simplified and expanded abstracts published in proceedings and book chapters. Studies related to neurodegenerative diseases, such as Huntington's disease and diabetes mellitus, were also excluded.

## RESULTS

A total of 13 articles were selected for the systematic review whose authorship, year of publication, study population, techniques used, and prominent results are shown in Table 1 ^11–23^.

**Table 1:** Bibliographic sources identified, type of exercise, technique used, sample, intervention, and outcomes.

No	Author	Animal population	Type of exercise	Techniques used	Results	Importance
1	Sable et al.^11^	Long-Evans Rat (n=28), Two groups (Exercise, Sedentary) DF-Exercise (n=7) DF-Sedentary (n=7) WD-Exercise (n=7) WD-Sedentary (n=7)	Treadmill: 25 m/min, three times/week for 30 min, six weeks	Tissue extraction and Western blotting	↑Protein BDNF, ARC, P-STAT3 in hippocampal	Exercise on Daniel Fast (DF) rats on spatial and neuroprotective working memory (WM) is more effective than on rats fed with the Western diet (WD)
2	O’Leary et al.^12^	Male Sprague Dawley Rat; Adult (8 weeks-old) and adolescent (4 weeks-old). n=41 (adolescent=20, adult=21) Two groups (Exercise) and (Sedentary control)	Running wheel Open field arena, diameter (90 cm) for seven weeks. Adult running; 3.96 km/day Adolescence running 3.43 km/day	qRT-PCR	↑Ekspresi mRNA ARC, Synaptophysin, BDNF, PSD-95, CREB, TLX, and DCX	Exercise initiated by adolescent rats (4 weeks) can increase mRNA expression as a marker of neural plasticity in the Hippocampus, whereas Exercise in adults does not increase gene expression. However, synaptophysin gene expression in the amygdala increased in adult rats (aged 8 weeks).
3	Sun et al.^13^	Sprague Dawley Rat; N=40 Pregnant Rat Disruptor di-(2-ethylhexyl)-phthalate (DEHP), Postnatal (days 22–56) Two groups (Sedentary, Exercise), Postnatal (days 57–65) (open field test, test, Morris Water Maze).	Treadmill. 8 m/min, 30 min/day, seven days/week for four weeks	Western Blott Ellisa	↑BDNF, NMDAR, ARC, and synaptophysin Protein Expression at Hippocampus.	Exercise on rats exposed to Disruptor di-(2-ethylhexyl)-phthalate (DEHP) postnatal days 22–56 and days 57–65 can restore learning and memory disorders.
4	Tsuchida et al.^14^	Adult male C57BL/6J Mice (4 weeks-old) n=56. At 8 weeks of age, mice; Two groups: Treadmill (n=16) and Rotarod (n=40).	Treadmill (15 m/min (T15) 30 min/day and Rotarod 30 rpm (R30)); diameter of the rod 3.2 cm for five days.	Immunohistochemistry	↑Protein c-Fos.	At an exercise intensity of 15 m/min (T15), it is more effective in increasing hippocampal nerve activation than in rotarod exercise at 30 rpm (R30).
5	Meireles et al.^15^	Male Wistar Rat (n=76); aged 2 and 22 months. Divided into five groups (Sedentary, Aerobic, Acrobatic, Resistance, and Combined)	Treadmill; Low intensity 5 m/min. Resistance training climbing (height 1 m, inclination of 85°) Acrobatic horizontal ladder (100 cm in diameter, 3 cm spaced rungs) Combined exercise modalities; 20-min exercise sessions, three times a week on alternate days, for 12 weeks	Chip	↑mRNA Promoter BDNF, c-Fos, and DNA methyltransferase 3a (Dnmt3a)	Exercise starting at the age of 22 months (aging) has an impact on cognitive decline. Resistance, acrobatic, aerobic, and combined modalities were able to enhance epigenetic (H3K9ac, H3K4me3, H4K8ac) and repressive (H3K9me2) marker mechanisms and aversive memory performance in aged mice
6	Schoenfeld et al.^16^	Adult male C57BL/6 Mice (n=24) (6 weeks of age) Divided in two groups (Exercise=12 and Sedentary=12)	Running wheel (swimming) 5 min, six weeks	Immunohistochemistry	Exercise ↑ protein c-Fos, ARC	In central dentate gyrus, exercise increased the number of new neurons and reduced anxiety behavior. In contrast, in the dorsal dentate gyrus, sedentary and exercised rats showed increased gene expression (c-Fos, ARC). (Interventioh rats experiencing stress while swimming in cold water
7	Keloglan et al.^17^	Male Wistar Rat; 3 weeks-old; Postnatal days (PNDs) 21–34. n=32 Control (C) n=8; Social Isolation (SI) n=8; Exercised (E) n=8; Social isolation + Exercise (SE) n=8.	Treadmill: direct current shock (0.1–0.15 mA), 20-60 min/days, five days per week, four weeks	RT-PCR	Exercise ↑NMDAR, mRNA Cdk5r, ASCL1. BDNF, Cdk5r, ARC, c-Fos	Long-term treadmill training enhances learning-related genes and neurogenesis without increasing cognitive behavior in socially isolated mice. Exercise can alter brain development, function, and development due to neuropsychiatric disorders.
8	Zielinski et al.^18^	Male C57BL/6J Mice; n=40 Normal sleep + Sedentary, Normal sleep + Exercise, Sleep restriction + Sedentary, and Sleep restriction + Exercise	Treadmill 18–21 m/min, 60 min/day, six days/week, 11 weeks and 5% grade.	Immunohistochemistry.	↑Protein c-Fos, BDNF	Exercise increased memory recall with increased c-Fos positive cells
9	Ransome and Hannan^19^	Female and male R6/1 HD transgenic mice, (12 weeks-old); Female wildtype (n=18) and R6/1 HD (n=18) 7 weeks of age control littermates; (n=9 wildtypes; n=9 R6/1 HD), run/enriched littermates (n=9 wildtypes; n=9 R6/1 HD)	Voluntary running 90 min, 7 days, 4 weeks	Immunofluorescence Immunohistochemistry	↑Protein c-Fos	Running induced Akt phosphorylation in the hippocampus of female wildtype mice, which was not reflected in R6/1 HD mice. Running in adult rats caused neurogenesis in the hippocampus
10	Zhong et al.^20^	Male C57BL/6 Mice; n=294, groups of 12–20 mice	Swimming, water depth 5–15 cm. 5 min, four times/session, every session 30 min, five days/week, four weeks	Immunohistochemistry	↑Protein c-Fos, histone acetylation, CREB) - binding protein (CBP)	There was increased hippocampal acetylation of H3K9, H4K5, and H4K12, an increased number of c-Fos-positive cells one hour after CFC training, and less memory impairment and increased histone acetylation and (CREB)-binding protein (CBP) in rats that underwent exercise regular swimming
11	Cefis et al.^21^	Wistar Rat, 10-week-old; n=69 Two groups: SED (sedentary), Exercise (EX12, EX18)	Treadmill Low-intensity exercise (12 m/min, EX12), High-intensity (18 m/min, EX18), 30 min/day, one week	Western Blotting	↑Protein c-Fos, BDNF, Synaptophysin	High-intensity exercise (E18) improves memory performance compared to low intensity (EX12) and sedentary.
12	Tsai et al.^22^	6-week-old Mice; n=30 Four groups: 1-day single-bout (Acute Exercise); Without Exercise (Basal); Exercise for one hour (E1h); and Exercise for one hour and rest at the home cage for two hours (E1hR2h)	Treadmill - Acute exercise; 10 m/min, 60 min - Long-time exercise: 10 m/min for 20–60 min/day, five days/week, four weeks	Immunohistochemistry Western Blott	↑Protein c-Fos	Acute exercise caused increased c-Fos+ cell density in the ventral hip hippocampal CA1 region, primary somatosensory cortex, other hippocampal subregions, and striatum Long-term exercise increased c-Fos+ cell density in the striatum, primary somatosensory, primary and secondary motor cortex, hippocampal subregion, hypothalamic nucleus, and lateral periaqueductal gray
13	Belviranlı and Okunda^23^	Female Wistar rats (aged 3 months) and (aged 20 months); n= 30 Four groups: Control (Yc, n=8); Young Exercise (Yt, n=8); Aged control (AC, n=7); and Aged Exercise (AT, n=7).	Running wheel, 90 days	Western Blott	↑mRNA BDNF, FNDC5, PGC-1α, mTOR, ARC, c-Fos, ERK, SIRT, dan FOXO	Exercise training improved spatial learning and memory in aged rats

Abbreviations: DF, Daniel Fast; WM, working memory; WD, Western diet; BDNF, brain-derived neurotrophic factor; CREB, cAMP-responsive element-binding protein; mRNA, messenger RiboNucleic Acid; Yc, Young control; Yt, Young Training; AT, Aged Training; CFC, contextual fear conditioning.

### Outcomes

**Figure 2:**
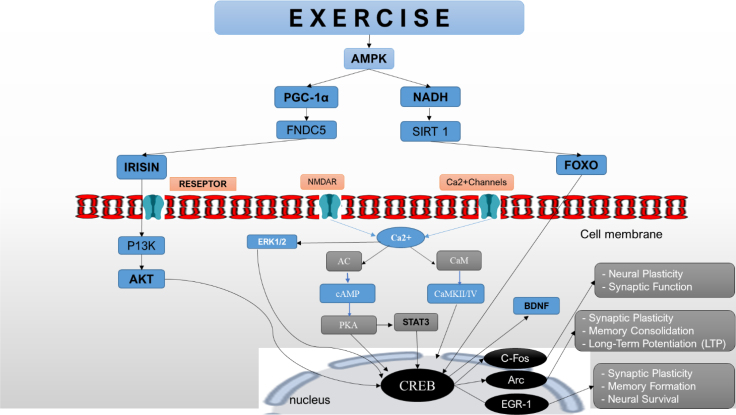
C-Fos, ARC, EGR-1 expression during exercise: physical exercise triggers physiological changes in the body by myokine expression in the muscles. Activation of membrane receptors in the brain: the neurotransmitters released bind to specific receptors in the brain, including in the hippocampus. This activation causes a cascade of intracellular events. Opening signaling pathways: the binding of neurotransmitters to their receptors initiates signaling pathways within hippocampal neurons. The cyclic adenosine monophosphate (cAMP) pathway is involved, CaMkII/IV. The cAMP pathway: activation of the cAMP pathway triggers a cascade of intracellular reactions, activating protein kinase A (PKA). PKA is an enzyme that phosphorylates and activates other proteins in gene expression. Once activated, PKA translocates to the nucleus of hippocampal neurons, where it phosphorylates and activates transcription factors such as CREB. Gene expression: CREB binds to a specific DNA sequence called the cAMP response element (CRE) in the promoter region of the target gene. This binding initiates a transcription of these genes directly to the start gene (c-Fos, ARC, EGR-1). Exercise stimulates neurotransmitter release, activates signaling pathways, phosphorylates transcription factors, and leads to c-Fos, ARC, and Egr-1 expression in the hippocampus. The activation of this gene indicates increased neural activity, synaptic plasticity, and cognitive benefits associated with exercise.

C-Fos, ARC, and EGR-1/zif268 are induced in neurons during neural activity in the hippocampus, including Morris’ water maze^24–27^. Physical activity upregulates neurotrophins and neuropeptides^28–30^ in long-term hippocampal potentiation (LTP)^31^. Molteni et al. (2002) showed in research that physical exercise increases hippocampal gene expression, including IEGs, associated with neuronal plasticity^32^ (Figure 2).

#### c-Fos expression during exercise

c-Fos is one of the first neuronal transcription genes whose induction is activity-dependent^33^ due to cAMP and Ca2+ stimulation by activating the CREB complex^34^. Increased c-Fos expression is an indicator of neural activation, as demonstrated by behavioral training in learning^35^, cognitive performance^36,37^, and memory formation^38^.

Physical exercise induces c-Fos expression in the rat hippocampal central nervous system, contributing to neuroplasticity^35,39^, increased neural activity^40^, and spatial memory coding^41^. c-Fos expression also plays a role in autonomic and somatomotor control and extends to other parts of the brain^42^. Additio ally, physical exercise increases the c-Fos expression through serum insulin-like growth factor-I (IGF-I) as a neuroprotective factor^43^.

Previous studies showed that physical exercise can improve cognitive function by increasing hippocampal neurogenesis^44^. This is supported by evidence from voluntary wheel running exercise, which maintains a stable level of neurogenesis through increased progenitor cell differentiation, mediated by elevated c-Fos levels in the dentate gyrus of rats^45^. Alongside c-Fos, the expression of EGR-1/zif268 and ARC also increases, further promoting neurogenesis activity in the hippocampal granule cell layer^46^. Voluntary running exercise induction also boosts c-Fos expression through Akt phosphorylation in the female rat hippocampus, enhancing adult hippocampal neurogenesis for cell survival, even in rats with Huntington's disease^19^. The expression of c-Fos also increases after treadmill exercise induction, influencing learning and spatial memory^18^. Cold water swimming is associated with increased activation of hippocampal interneurons and a higher number of new neurons in the dentate gyrus^16^.

Grinspun et al. research in 2019^47^ explained that running wheel exercise improves memory, as evaluated by increased c-Fos immunoreactivity in the tuberomammillary nucleus (TMN). Similarly, treadmill exercise is shown to benefit several neurodegenerative diseases, such as Alzheimer's, by increasing c-Fos expression in the hippocampus in mice after streptozotocin (STZ) injection, reducing long-term memory deficits^48^. In rats with diabetes induced by STZ injection, subsequent treadmill exercise can enhance neuroplasticity and spatial memory by upregulating the expression of the c-Fos nerve gene in the hippocampus^49^. Increased c-Fos expression resulting from physical exercise and therapy aims to boost neural activity, learning, and memory, even in children born to alcoholic mothers during pregnancy, thus countering the effects of alcohol poisoning on postpartum mothers and their babies^50^. Swimming exercise has been found to mitigate isoflurane-induced memory impairment by increasing the expression of c-Fos, a CREB-binding protein (CBP), which elevates hippocampal histone acetylation and activates more neuron cells during memory formation, ultimately improving memory impairment^20^.

Exercise intensity plays a significant role in c-Fos expression. High-intensity treadmill exercise increases c-Fos expression and brain-derived neurotrophic factor (BDNF) levels in the hippocampus, thereby enhancing memory through heightened neuroplasticity in both the hippocampus and prefrontal cortex^21^. Foley et al. study also demonstrated that the induction of c-Fos mRNA expression at high intensities significantly increases c-Fos mRNA activation in the hippocampus, affecting the pattern of potential brain activation compared to low-intensity exercise^51^. Low-intensity acute and long-term treadmill exercise, performed at a speed of 10 m/min over four weeks, induces c-Fos expression by increasing the density of c-Fos+ cells in various brain regions, including the striatum, primary somatosensory and secondary motor cortex, hippocampal subregion, nucleus hypothalamus, and lateral periaqueductal grey. This positive correlation with BDNF expression suggests that long-term exercise has a broader regional and temporal impact on the brain than acute exercise^22^. Moderate-intensity treadmill exercise has proven more efficient in activating hippocampal nerves than rotarod exercise^14^.

In addition to exercise intensity, the type of exercise also significantly influences c-Fos expression in the hippocampus, especially in treadmill and rotarod exercises. Immunohistochemical examination of c-Fos as a nerve activation marker revealed that treadmill exercise at 15 m/min (T15) significantly increases c-Fos expression in all hippocampus subfields, including CA3, CA1, and the dentate gyrus. In contrast, 30 rpm rotarod exercise (R30) does not result in increased c-Fos expression^14^.

The duration of exercise in voluntary wheel running over 25 days is found to increase the survival of new neurons and affect c-Fos expression. Longer exercise duration has a greater impact on the rate of neurogenesis, as it leads to increased differentiation of hippocampal progenitor cells in adult rat models^52^. The expression of c-Fos induced by physical exercise is influenced by exercise intensity, type, and duration, with higher intensity and longer durations resulting in increased c-Fos expression in the hippocampus^53,54^.

Increased c-Fos expression in the rat hippocampus due to treadmill exercise was observed across all age groups. The cornu ammonis (CA) region showed the highest increase in c-Fos expression with treadmill exercise in 4-week-old rats, while in the dentate gyrus, the highest increase was observed in rats aged 62 weeks. This suggests that age is a crucial factor in regulating c-Fos expression in the hippocampus, as it appears to be age-dependent when induced by exercise^55^.

#### Expression ARC during exercise

ARC is one of the most characteristic molecules that play a key role in memory formation. This gene encodes proteins involved in synaptic functions related to serotonin, glutamate, and dopamine, distinguishing it from c-Fos and EGR-1^56–59^. ARC expression is regulated by EGR-1^60^ and its mRNA is transported to dendrites^61,62^, making it a marker of neural activity^56,63^.

Under certain conditions, mRNA ARCs can form in postsynaptic dendrites independently of presynaptic axons^64,65^. ARC acts on both new and established synapses, playing a crucial role in mechanisms related to synaptic plasticity, such as long-term potentiation (LTP) and long-term depression (LTD)^3,66^. ARC encodes an F-actin-associated (ARC)^10,67^ growth factor that contributes to dendritic reconfiguration^62,68^. Therefore, both the ARC and EGR-1 genes, albeit to varying degrees, influence changes in neural tissue and play more specific roles than c-Fos^69^.

Chronic aerobic exercise improves working memory and can be evaluated as a neuroprotective measure due to increased ARC and BDNF expression in the hippocampus. This effect was observed in rats subjected to a Western diet combined with a plant-based Daniel Fast (DF) intervention^11^. The expression of ARC induced by physical exercise is influenced by age, with physical exercise during adolescence increasing the expression of genes related to synaptic plasticity and cognitive function in the hippocampus, including synaptophysin, BDNF, PSD-95, CREB, ARC, TLX, and DCX. However, physical exercise in adulthood does not affect the expression of these genes, suggesting that exercise during adolescence is more effective in enhancing cognitive function^12^. This finding was supported by Sun et al. research in 2021^13^ whose research explained that treadmill exercise can improve neuroplasticity in male rats exposed to (2-ethylhexyl)-phthalate DEHP before birth, leading to memory and spatial learning deficits during late adolescence. Exercise during childhood and adolescence can restore gene expression and improve learning and memory deficits^16^. In old age, wheel running exercise increases the expression of genes such as BDNF, FNDC5, PGC-1α, mTOR, ARC, c-Fos, ERK, SIRT, and FOXO. This can help counter cognitive dysfunction associated with aging, which is characterized by decreased expression of genes and proteins like PGC-1α, FNDC5, and BDNF in the hippocampus^23^.

#### Expression EGR-1 during exercise

EGR-1, known by various names such as zif268, NGFI-A, Krox 24, or ZENK^70,71^, is a transcription factor whose expression is induced by various factors, including injury, stress, cell differentiation, and extracellular signals such as growth factors, neurotransmitters, and peptides^72–74^. EGR-1 expression exhibits a distinct pattern in the brain compared to c-Fos^68–75^. It plays a crucial role in mediating the expression of multiple genes involved in neural processes, ranging from growth control to changes in synaptic plasticity^76–78^. EGR-1 is relatively highly expressed during neural activity^72,79^, particularly in the hippocampus^3,80^ and the dentate gyrus^7^. Its role in learning and memory is attributed to its modulation of synaptic plasticity, including the remodeling of dendrites, and synapses, and the formation of new synaptic connections^69^.

This study supports the role of EGR-1 in learning and memory formation, affecting neural and cognitive functions. EGR-1 functions as a transcription factor that regulates numerous identified target genes. In contrast to c-Fos, which primarily targets genes related to vesicular transport and neurotransmitter release, EGR-1 targets genes that often depend on clathrin or actin processes^81,82^.

Physical exercise also influences EGR-1 expression, with different durations of exercise affecting EGR-1 expression in the hippocampus^83^. However, research on the effects of exercise on EGR-1 expression is still limited, and conclusive findings on its impact in the hippocampus cannot be drawn at this time.

## DISCUSSION

The review analysis above demonstrates that environment and exercise influence the expression of EGR-1, ARC, and c-Fos genes in memory formation and storage. C-Fos gene expression serves as an indicator of neuronal activity. When neurons are active, the c-Fos gene becomes activated, leading to increased production of c-Fos protein. This protein plays a crucial role in neuroplasticity, involving structural and functional changes in the brain. Its role in the hippocampus has been investigated in mice, where c-Fos is involved in neurogenesis and the significance of the AP-1 transcription factor in c-Fos development^84^. The activation of the c-Fos gene and protein occurs rapidly after stimulation and depends on the type and timing of the stimulus. C-Fos requires the participation of other genes, such as c-Jun, for its expression during heterodimer formation. C-Fos dimerizes with the c-Jun protein to form the AP-1 factor, which promotes the transcription of various genes. While the production and removal of c-Fos are part of cellular homeostasis, overexpression can lead to increased cell proliferation^85^.

Similarly, ARC expression is influenced by physical exercise in the hippocampus. When we engage in physical exercise, synaptic activity in the hippocampus increases, facilitating communication between neurons. This heightened synaptic activity triggers the expression of the ARC gene. The primary role of ARC protein is in the process of neuroplasticity. When the ARC gene is activated, ARC proteins are produced and move to the synapses, where neurons communicate. ARC protein acts as a bridge between synaptic signaling and structural changes in neurons, regulating synaptic changes and promoting the formation and maintenance of new synaptic pathways^86^.

In the context of the hippocampus, exercise-induced c-Fos and ARC gene expression may facilitate synaptic changes that support learning and memory. The ARC protein also plays a role in memory consolidation, converting short-term memory into long-term memory. Additionally, both c-Fos and ARC proteins are involved in the storage and reactivation of existing memories^66,87^. Therefore, increased ARC gene expression due to physical exercise can influence neuroplasticity in the hippocampus, including memory formation and maintenance. Research has also demonstrated that a deficiency in the ARC gene can impair cognitive performance and memory, underscoring the critical role of this gene in brain function^88^.

ARC expression is regulated by EGR-1^60^. EGR-1 is a transcription factor involved in gene regulation in cells, and its activation can affect the expression of genes involved in synaptic plasticity, learning, and memory in the hippocampus. EGR-1 gene expression plays a pivotal role in the brain's response to physical exercise in the hippocampus. EGR-1 is a transcription factor activated by external stimuli, including physical exercise. Physical exercise is shown to increase EGR-1 gene expression in the hippocampus. During physical exercise, there is an increase in synaptic activity and the release of neurotransmitters in the hippocampus, resulting in an external stimulus that triggers the activation of the EGR-1 gene^46^.

The primary function of the EGR-1 protein is to regulate the expression of other genes in response to stimuli. The EGR-1 protein acts as a transcription factor, binding to the promoter of the target gene and regulating the production of that protein. In the context of the hippocampus, exercise-induced increased expression of the EGR-1 gene influences other genes involved in synaptic plasticity, neurogenesis, and cognitive function^82^.

Although numerous studies demonstrated a relationship between physical exercise, c-Fos, ARC, EGR-1 gene expression, and hippocampal function, this area remains a subject of ongoing research. In particular, research into the effect of exercise on EGR-1 gene and protein expression is still limited. Therefore, further research is needed to understand the impact of physical exercise on EGR-1 gene expression, hippocampal function, and other factors such as the type of exercise, intensity, and duration, which can also influence the brain's response.

In conclusion, the expression of IEG genes, including c-Fos, EGR-1, and ARC, in hippocampal neurons increases after being induced by exercise. This beneficial process in synaptic plasticity is associated with learning, memory, and neurogenesis.

## References

[1] Yochiy A, Britto LRG, Hunziker MHL (2012). Novelty, but not operant aversive learning, enhances Fos and Egr-1 expression in the medial prefrontal cortex and hippocampal areas of rats. Behav Neurosci.

[2] Kim S, Kim H, Um JW (2018). Synapse development organized by neuronal activity-regulated immediate-early genes. Exp Mol Med.

[3] Minatohara K, Akiyoshi M, Okuno H (2016). Role of immediate-early genes in synaptic plasticity and neuronal ensembles underlying the memory trace. Front Mol Neurosci.

[4] Loebrich S, Nedivi E (2009). The function of activity-regulated genes in the nervous system. Physiol Rev.

[5] Kandel ER (2012). The molecular biology of memory: cAMP, PKA, CRE, CREB-1, CREB-2, and CPEB. Mol Brain.

[6] Wisden W, Errington ML, Williams S, Dunnett SB, Waters C, Hitchcock D (1990). Differential expression of immediate early genes in the hippocampus and spinal cord. Neuron.

[7] Worley PF, Christy BA, Nakabeppu Y, Bhat RV, Cole AJ, Baraban JM (1991). Constitutive expression of zif268 in neocortex is regulated by synaptic activity. Proc Natl Sci U S A.

[8] Farris S, Lewandowski G, Cox CD, Steward O (2014). Selective localization of arc mRNA in dendrites involves activity- and translation-dependent mRNA degradation. J Neurosci.

[9] Na Y, Park S, Lee C, Kim DK, Park JM, Sockanathan S (2016). Real-Time imaging reveals properties of glutamate-induced Arc/Arg 3.1 translation in neuronal dendrites. Neuron.

[10] Abraham WC, Dragunow M, Tate WP (1991). The role of immediate early genes in the stabilization of long-term potentiation. Mol Neurobiol.

[11] Sable HJ, MacDonnchadh JJ, Lee HW, Butawan M, Simpson RN, Krueger KM (2022). Working memory and hippocampal expression of BDNF, ARC, and P-STAT3 in rats : effects of diet and exercise. Nutr Neurosci.

[12] O’Leary JD, Hoban AE, Cryan JF, O’Leary OF, Nolan YM (2019). Differential effects of adolescent and adult-initiated voluntary exercise on context and cued fear conditioning. Neuropharmacology.

[13] Sun GC, Lee YJ, Lee YC, Yu HF, Wang DC (2021). Exercise prevents the impairment of learning and memory in prenatally phthalate-exposed male rats by improving the expression of plasticity-related proteins. Behav Brain Res.

[14] Tsuchida R, Yamaguchi T, Funabashi D, Koumi Y, Kita I, Nishijima T (2022). Exercise type influences the effect of an acute bout of exercise on hippocampal neuronal activation in mice. Neurosci Lett.

[15] Meireles LCF, Galvão F, Walker DM, Cechinel LR, Grefenhagen AIS, Andrade G (2019). Exercise modalities improve aversive memory and survival rate in aged rats : role of hippocampal epigenetic modifications. Mol Neurobiol.

[16] Schoenfeld TJ, Rada P, Pieruzzini PR, Hsueh B, Gould E (2013). Physical exercise prevents stress-induced activation of granule neurons and enhances local inhibitory mechanisms in the dentate gyrus. J Neurosci.

[17] Keloglan S, Sahin L, Cevik OS (2019). Long-term treadmill exercise upregulated hippocampal learning-related genes without improving cognitive behaviour in socially isolated rats. Folia Morphol (Warsz).

[18] Zielinski MR, Davis JM, Fadel JR, Youngstedt SD (2013). Influence of chronic moderate sleep restriction and exercise training on anxiety, spatial memory, and associated neurobiological measures in mice. Behav Brain Res.

[19] Ransome MI, Hannan AJ (2013). Impaired basal and running-induced hippocampal neurogenesis coincides with reduced Akt signaling in adult R6/1 HD mice. Mol Cell Neurosci.

[20] Zhong T, Ren F, Huang CS, Zou WY, Yang Y, Pan YD (2016). Swimming exercise ameliorates neurocognitive impairment induced by neonatal exposure to isoflurane and enhances hippocampal histone acetylation in mice. Neuroscience.

[21] Cefis M, Prigent-Tessier A, Quirié A, Pernet N, Marie C, Garnier P (2019). The effect of exercise on memory and BDNF signaling is dependent on intensity. Brain Struct Funct.

[22] Tsai SF, Wen YW, Kuo YM (2019). Acute and long-term treadmill running differentially induce c-Fos expression in region- and time- dependent manners in mouse brain. Brain Struct Funct.

[23] Belviranlı M, Okudan N (2018). Exercise training protects against aging-induced cognitive dysfunction via activation of the hippocampal PGC-1α/FNDC5/BDNF pathway. Neuromolecular Med.

[24] Vann SD, Brown MW, Erichsen JT, Aggleton JP (2000). Fos imaging reveals differential patterns of hippocampal and parahippocampal subfield activation in rats in response to different spatial memory tests. J Neurosci.

[25] Guzowski JF, Miyashita T, Chawla MK, Sanderson J, Maes LI, Houston FP (2006). Recent behavioral history modifies coupling between cell activity and Arc gene transcription in hippocampal CA1 neurons. Proc Natl Acad Sci U S A.

[26] Hall J, Thomas KL, Everitt BJ (2001). Cellular imaging of zif268 expression in the hippocampus and amygdala during contextual and cued fear memory retrieval : selective activation of hippocampal CA1 neurons during the recall of contextual memories. J Neurosci.

[27] Mamiya N, Fukushima H, Suzuki A, Matsuyama Z, Homma S, Frankland PW (2009). Brain region-specific gene expression activation required for reconsolidation and extinction of contextual fear memory. J Neurosci.

[28] Bucinskaite V, Theodorsson E, Crumpton K, Stenfors C, Ekblornf A, Lundeberg T (1996). Effects of repeated sensory stimulation (electro-acupuncture) and physical exercise (running) on open-field behaviour and concentrations of neuropeptides in the hippocampus in WKY and SHR rats. Eur J Neurosci.

[29] Russo-Neustadt A, Beard RC, Cotman CW (1999). Exercise, antidepressant medications, and enhanced brain derived neurotrophic factor expression. Neuropsychopharmacology.

[30] Neeper SA, Gómez-Pinilla F, Choi J, Cotman C (1995). Exercise and brain neurotrophins. Nature.

[31] Chen G, Kolbeck R, Barde YA, Bonhoeffer T, Kossel A (1999). Relative contribution of endogenous neurotrophins in hippocampal long-term potentiation. J Neurosci.

[32] Molteni R, Ying Z, Gómez-Pinilla F (2002). Differential effects of acute and chronic exercise on plasticity-related genes in the rat hippocampus revealed by microarray. Eur J Neurosci.

[33] Morgan J, Curran T (1988). Calcium as a modulator of the immediate-early gene cascade in neurons. Cell Calcium.

[34] Pennypacker K (1998). Ap-1 transcription factors: short- and long-term modulators of gene expression in the brain. Int Rev Neurobiol.

[35] Tischmeyer W, Grimm R (1999). Activation of immediate early genes and memory formation. Cell Mol Life Sci.

[36] Radulovic J, Kammermeier J, Spiess J (1998). Relationship between fos production and classical fear conditioning : effects of novelty, latent inhibition, and unconditioned stimulus preexposure. J Neurosci.

[37] Bertaina-Anglade V, Tramu G, Destrade C (2000). Differential learning-stage dependent patterns of c-Fos protein expression in brain regions during the acquisition and memory consolidation of an operant task in mice. Eur J Neurosci.

[38] Lopez LM, Harris SE, Luciano M, Liewald D, Davies G, Gow AJ (2012). Evolutionary conserved longevity genes and human cognitive abilities in elderly cohorts. Eur J Hum Genet.

[39] Hughes P, Lawlor P, Dragunow M (1992). Basal expression of Fos, Fos-related, Jun, and Krox 24 proteins in rat hippocampus. Brain Res Mol Brain Res.

[40] Bellchambers CE, Chieng B, Keay KA, Christie MJ (1998). Swim-stress but not opioid withdrawal increases expression of c-fos immunoreactivity in rat periaqueductal gray neurons which project to the rostral ventromedial medulla. Neuroscience.

[41] Guzowski JF (2002). Insights into immediate-early gene function in hippocampal memory consolidation using antisense oligonucleotide and fluorescent imaging approaches. Hippocampus.

[42] Liste I, Guerra MJ, Caruncho HJ, Labandeira-Garcia JL (1997). Treadmill running induces striatal Fos expression via NMDA glutamate and dopamine receptors. Exp Brain Res.

[43] Carro E, Nuñez A, Busiguina S, Torres-Aleman I (2000). Circulating insulin-like growth factor I mediates effects of exercise on the brain. J Neurosci.

[44] Yau SY, Gil-Mohapel J, Christie BR, So KF (2014). Physical exercise-induced adult neurogenesis : a good strategy to prevent cognitive decline in neurodegenerative diseases?. Biomed Res Int.

[45] Clark PJ, Brzezinska WJ, Thomas MW, Ryzhenko NA, Toshkov SA, Rhodes JS (2008). Intact neurogenesis is required for benefits of exercise on spatial memory but not motor performance or contextual fear conditioning in C57BL/6J mice. Neuroscience.

[46] Clark PJ, Bhattacharya TK, Miller DS, Rhodes JS (2011). Induction of c-Fos, Zif268, and Arc from acute bouts of voluntary wheel running in new and pre-existing adult mouse hippocampal granule neurons. Neuroscience.

[47] Grinspun N, Fuentealba Y, Falcon R, Valdés JL (2019). c-Fos expression in the ascending arousal system induced by physical exercise in rats: implication for memory performance. Brain Res.

[48] Jee YS, Ko IG, Sung YH, Lee JW, Kim YS, Kim SE (2008). Effects of treadmill exercise on memory and c-Fos expression in the hippocampus of the rats with intracerebroventricular injection of streptozotocin. Neurosci Lett.

[49] You JSH, Kim CJ, Kim MY, Byun YG, Ha SY, Han BS (2009). Long-term treadmill exercise-induced neuroplasticity and associated memory recovery of streptozotocin-induced diabetic rats: an experimenter blind, randomized controlled study. NeuroRehabilitation.

[50] Sim YJ, Kim H, Shin MS, Chang HK, Shin MC, Ko IG (2008). Effect of postnatal treadmill exercise on c-Fos expression in the hippocampus of rat pups born from the alcohol-intoxicated mothers. Brain Dev.

[51] Foley TE, Brooks LR, Gilligan LJ, Burghardt PR, Koch LG, Britton SL (2012). Brain activation patterns at exhaustion in rats that differ in inherent exercise capacity. PLoS One.

[52] Clark PJ, Kohman RA, Miller DS, Bhattacharya TK, Haferkamp EH, Rhodes JS (2010). Adult hippocampal neurogenesis and c-Fos induction during escalation of voluntary wheel running in C57BL/6J mice. Behav Brain Res.

[53] Lee TH, Jang MH, Shin MC, Lim BV, Kim YP, Kim H (2003). Dependence of rat hippocampal c-Fos expression on intensity and duration of exercise. Life Sci.

[54] Lee MH, Kim H, Lim BV, Chang HK, Lee TH, Jang MH (2003). Naloxone potentiates treadmill running-induced increase in c-Fos expression in rat hippocampus. Life Sci.

[55] Kim SH, Kim H, Kim SS, Shin MS, Chang HK, Lee TH The influence of age on the treadmil exercise-induced c-Fos expression in the hippocampus of rats. Neurosci Res.

[56] Granado N, Ortiz O, Suárez LM, Martín ED, Ceña V, Solís JM (2008). D1 but not D5 dopamine receptors are critical for LTP, spatial learning, and LTP-induced arc and zif268 expression in the hippocampus. Cereb Cortex.

[57] Karabeg MM, Grauthoff S, Kollert SY, Weidner M, Heiming RS, Jansen F (2013). 5-HTT deficiency affects neuroplasticity and increases stress sensitivity resulting in altered spatial learning performance in the Morris water maze but not in the Barnes maze. PloS One.

[58] Pastuzyn ED, Keefe KA (2014). Changes in neural circuitry regulating response-reversal learning and Arc-mediated consolidation of learning in rats with methamphetamine-induced partial monoamine loss. Neuropsychopharmacology.

[59] Mastwal S, Cao V, Wang KH (2016). Genetic feedback regulation of frontal cortical neuronal ensembles through activity-dependent arc expression and dopaminergic input. Front Neural Circuits.

[60] Li L, Carter J, Gao X, Whitehead J, Tourtellotte WG (2005). The neuroplasticity-associated arc gene is a direct transcriptional target of early growth response (Egr) transcription factors. Mol Cell Biol.

[61] Fujimoto T, Tanaka H, Kumamaru E, Okamura K, Miki N (2004). Arc interacts with microtubules/microtubule-associated protein 2 and attenuates microtubule-associated protein 2 immunoreactivity in the dendrites. J Neurosci Res.

[62] Steward O, Worley PF (2001). A cellular mechanism for targeting newly synthesized mRNAs to synaptic sites on dendrites. Proc Natl Acad Sci U S A.

[63] Shepherd JD, Rumbaugh G, Wu J, Chowdhury S, Plath N, Kuhl D (2006). Arc/Arg3.1 mediates homeostatic synaptic scaling of AMPA receptors. Neuron.

[64] Moga DE, Calhoun ME, Chowdhury A, Worley P, Morrison JH, Shapiro LM (2004). Activity-regulated cytoskeletal-associated protein is localized to recently activated excitatory synapses. Neuroscience.

[65] Dynes JL, Steward O (2012). Arc mRNA docks precisely at the base of individual dendritic spines indicating the existence of a specialized microdomain for synapse-specific mRNA translation. J Comp Neurol.

[66] Korb E, Finkbeiner S (2011). Arc in synaptic plasticity : from gene to behavior. Trends Neurosci.

[67] Guzowski JF, Lyford GL, Stevenson GD, Houston FP, McGaugh JL, Worley PF (2000). Inhibition of activity-dependent arc protein expression in the rat hippocampus impairs the maintenance of long-term potentiation and the consolidation of long-term memory. J Neurosci.

[68] Pinaud R (2004). Experience-dependent immediate early gene expression in the adult central nervous system : evidence from enriched-environment studies. Int J Neurosci.

[69] Gallo FT, Katche C, Morici JF, Medina JH, Weisstaub NV (2018). Immediate early genes, memory and psychiatric disorders: Focus on c-Fos, Egr1 and Arc. Front Behav Neurosci.

[70] Milbrandt J (1987). A nerve growth factor-induced gene encodes a possible transcriptional regulatory factor. Science.

[71] Lemaire P, Relevant O, Bravo R, Charnay P (1988). Two mouse genes encoding potential transcription factors with identical DNA-binding domains are activated by growth factors in cultured cells. Proc Natl Acad Sci U S A.

[72] Herdegen T, Leah JD (1998). Inducible and constitutive transcription factors in the mammalian nervous system: control of gene expression by Jun, Fos and Krox, and CREB/ATF proteins. Brain Res Brain Res Rev.

[73] O’Donovan KJ, Tourtellotte WG, Milbrandt J, Baraban JM (1999). The EGR family of transcription-regulatory factors: progress at the interface of molecular and systems neuroscience. Trends Neurosci.

[74] Clements KM, Wainwright PE (2010). Swim stress increases hippocampal Zif268 expression in the spontaneously hypertensive rat. Brain Res Bull.

[75] Mack K, Day M, Milbrandt J, Gottlieb DI (1990). Localization of the NGFI-A protein in the rat brain. Brain Res Mol Brain Res.

[76] Williams JM, Beckmann AM, Mason-Parker SE, Abraham WC, Wilce PA, Tate WP (2000). Sequential increase in Egr-1 and AP-1 DNA binding activity in the dentate gyrus following the induction of long-term potentiation. Brain Res Mol Brain Res.

[77] Bozon B, Davis S, Laroche S (2002). Regulated transcription of the immediate-early gene Zif268: mechanisms and gene dosage-dependent function in synaptic plasticity and memory formation. Hippocampus.

[78] Maddox SA, Monsey MS, Schafe GE (2010). Early growth response gene 1 (Egr-1) is required for new and reactivated fear memories in the lateral amygdala. Learn Mem.

[79] Beckmann AM, Matsumoto I, Wilce PA (1997). AP-1 and Egr DNA-binding activities are increased in rat brain during ethanol withdrawal. J Neurochem.

[80] Desjardins S, Mayo W, Vallée M, Hancock D, Le Moal M, Simon H (1997). Effect of aging on the basal expression of c-Fos, c-Jun, and Egr-1 proteins in the hippocampus. Neurobiol Aging.

[81] Koldamova R, Schug J, Lefterova M, Cronican AA, Fitz NF, Davenport FA (2014). Genome-wide approaches reveal EGR1-controlled regulatory networks associated with neurodegeneration. Neurobiol Dis.

[82] Duclot F, Kabbaj M (2017). The role of early growth response 1 (EGR1) in brain plasticity and neuropsychiatric disorders. Front Behav Neurosci.

[83] Salame S, Garcia PC, Real CC, Borborema J, Mota-Ortiz SR, Britto LRG (2016). Distinct neuroplasticity processes are induced by different periods of acrobatic exercise training. Behav Brain Res.

[84] Velazquez FN, Caputto BL, Boussin FD (2015). c-Fos importance for brain development. Aging (Albany NY).

[85] Lara Aparicio SY, Laureani Fierro AJ, Aranda Abreu GE, Toledo Cárdenas R, García Hernández LI, Coria Ávila GA (2022). Current opinion on the use of c-Fos in neuroscience. NeuroSci.

[86] Nikolaienko O, Patil S, Eriksen MS, Bramham CR (2018). Arc protein: a flexible hub for synaptic plasticity and cognition. Semin Cell Dev Biol.

[87] Gandolfi D, Cerri S, Mapelli J, Polimeni M, Tritto S, Fuzzati-Armentero MT (2017). Activation of the CREB/c-Fos pathway during long-term synaptic plasticity in the cerebellum granular layer. Front Cell Neurosci.

[88] Shepherd JD, Bear MF (2011). New views of Arc, a master regulator of synaptic plasticity. Nat Neurosci.

